# Asymptomatic recrudescence after artemether–lumefantrine treatment for uncomplicated falciparum malaria: a systematic review and meta-analysis

**DOI:** 10.1186/s12936-020-03520-1

**Published:** 2020-12-09

**Authors:** Rida Mumtaz, Lucy C. Okell, Joseph D. Challenger

**Affiliations:** 1 Faculty of Medicine, Imperial College London, London, UK; 2 Medical Research Council Centre for Global Infections Disease Analysis, Department of Infectious Disease Epidemiology, Imperial College London, London, UK

**Keywords:** Malaria, *Plasmodium falciparum*, Artemisinin-based combination therapy, Artemether–lumefantrine, Clinical trials, Treatment failure, Transmission, Systematic review

## Abstract

**Background:**

In clinical trials of therapy for uncomplicated *Plasmodium falciparum,* there are usually some patients who fail treatment even in the absence of drug resistance. Treatment failures, which can be due to recrudescence or re-infection, are categorized as ‘clinical’ or ‘parasitological’ failures, the former indicating that symptoms have returned. Asymptomatic recrudescence has public health implications for continued malaria transmission and may be important for the spread of drug-resistant malaria. As the number of recrudescences in an individual trial is often low, it is difficult to assess how commonplace asymptomatic recrudescence is, and with what factors it is associated.

**Methods:**

A systematic literature review was carried out on clinical trials of artemether-lumefantrine (AL) in patients seeking treatment for symptomatic uncomplicated falciparum malaria, and information on symptoms during treatment failure was recorded. Only treatment failures examined by polymerase chain reaction (PCR) were included, so as to exclude re-infections. A multivariable Bayesian regression model was used to explore factors potentially explaining the proportion of recrudescent infections which are symptomatic across the trials included in the study.

**Results:**

Across 60 published trials, including 9137 malaria patients, 37.8% [95% CIs (26.6–49.4%)] of recrudescences were symptomatic. A positive association was found between transmission intensity and the observed proportion of recrudescences that were asymptomatic. Symptoms were more likely to return in trials that only enrolled children aged < 72 months [odds ratio = 1.62, 95% CIs (1.01, 2.59)]. However, 84 studies had to be excluded from this analysis, as recrudescences were not specified as symptomatic or asymptomatic.

**Conclusions:**

AL, the most widely used treatment for uncomplicated *P. falciparum* in Africa, remains a highly efficacious drug in most endemic countries. However in the small proportion of patients where AL does not clear parasitaemia, the majority of patients do not develop symptoms again and thus would be unlikely to seek another course of treatment. This continued asymptomatic parasite carriage in patients who have been treated may have implications for drug-resistant parasites being introduced into high-transmissions settings.

## Background

Artemisinin combination therapy (ACT), the World Health Organization-recommended treatment for uncomplicated *Plasmodium falciparum*, remains highly effective in Africa [1]. ACT requires a 3-day dosing regimen, with either one or 2 doses a day [2]. However, even if dosing and patient adherence to treatment is optimal, as occurs in clinical trials with directly observed treatment, the treatment will fail to fully clear parasites in a small proportion of patients, usually under 5% [3]. Pharmacokinetic studies of drug treatment demonstrate that the inter-individual variation in the absorption and elimination of drugs is very large, and has been linked to treatment outcome in a number of studies [4–8]. There is evidence that severe acute malnutrition can inhibit drug absorption [9], and that pharmacogenetic factors play a role [10, 11]. In routine healthcare settings, additional factors can cause treatment to fail, such as imperfect adherence to the dosing regimen [12, 13]. Some front-line drugs, such as lumefantrine, require that doses be taken with fatty food or drink to ensure optimal absorption [14].

Treatment failures have a number of undesirable consequences: most immediately for the patient concerned, who may feel better initially but become symptomatic again as the parasites which have survived treatment multiply once more. In addition, the patient may be able to infect a feeding mosquito thus contributing to malaria transmission. Furthermore, a parasite population being exposed to, but not completely cleared by, anti-malarial drugs has implications for the development of drug resistance. During the evolution and spread of drug resistance, drug-sensitive parasites coexist with more resistant parasites. Some patients have genetically distinct sub-populations of parasites within their infection, either because mutations have occurred de novo as the parasites multiplied during the current infection, or because mosquitoes have injected more than one parasite clone in a single or multiple bites. If there are any parasites with some degree of resistance to the drug within a patient’s infection, they have a survival advantage over more sensitive parasites. Those which survive treatment are more likely to be transmitted to others. In this regard, an important consideration is whether patients who have failed treatment become symptomatic again. The reoccurrence of symptoms, although unpleasant for patients, may have positive implications at the community level. If a patient’s recrudescent infection is treated again, the capacity for further transmission is diminished [15]. Even if parasites are partially resistant, the treatment has a chance of being successful the second time, since very few types of drug resistance confer 100% lack of susceptibility to drugs. Although patients who do not become symptomatic after the infection recrudesces may be identified and retreated in a clinical trial, this is very unlikely in routine healthcare settings. Untreated infections of falciparum malaria can be very long lasting [16, 17], during which time it may be possible to infect feeding mosquitoes.

One can gain insight into the proportion of treatment failures that become symptomatic from clinical trials. Typically, treatment failures that are recorded in a trial are stratified into different categories. Early treatment failures (ETFs) indicate that the patient did not respond to treatment, and falls into one of 4 categories: (i) indications of severe malaria on day 1, 2 or 3 in a patient who is still parasite positive; (ii) parasitaemia on day 2 that is higher than observed on admission; (iii) parasitaemia on day 3 with axilliary temperature ≥ 37.5 °C; (iv) parasitaemia on day 3 ≥ 25% of that observed on admission [18]. Late treatment failures (LTFs) indicate that although patients responded to treatment and clinical symptoms abated, the drug treatment did not completely clear the parasite population, although the parasite density may become too low to be detectable by microscopy. If the infection is not cleared before drug concentrations decline below effective concentrations, the parasite population can grow in size again. For ACT, LTFs are rarely observed in the first 2 weeks (e.g., [19]), and patients in clinical trials should be followed up for at least 28 days [18]. Late parasitological failures (LPFs) indicate that patients were found to be parasite-positive during follow-up without the presence of symptoms (axilliary temperature < 37.5 °C). Late clinical failures (LCFs) indicate that patients were symptomatic (indications of severe malaria or an axilliary temperature ≥ 37.5 °C) and positive for parasites [18]. Infections detected during the follow-up period of a trial should be evaluated by polymerase chain reaction (PCR), to discriminate between a recrudescent infection and an infection acquired subsequently. In an individual trial, the number of PCR-corrected LTFs is often very small [3], which means that it is hard to gain insight into the likelihood of symptoms returning after parasitaemia rebounds. As only PCR-corrected LTFs are being considered here, the terms ‘symptomatic recrudescences’ and ‘asymptomatic recrudescences’ shall be used to refer respectively to LCFs and LPFs due to recrudescent infections. The definition of an LCF (given above) does not encompass all symptoms caused by a malaria infection (e.g., chills, headache, malaise, cough, diarrhoea [20]), but here an LPF caused by a recrudescent infection will be referred to as an asymptomatic recrudescence throughout.

Here, a systematic review of clinical trials was conducted to quantify the proportion of PCR-corrected LTFs that were recorded as being symptomatic failures (symptomatic recrudescences). Intuitively, this proportion will depend on the drug treatment administered: drugs that are eliminated more slowly from the body should suppress parasite densities for a longer period of time. Therefore, the systematic review is restricted to clinical trials of a single therapy, the ACT artemether-lumefantrine (AL). As of 2017, AL is a first- or second-line treatment for uncomplicated falciparum malaria in 59 countries across the world, including 32 in Africa [21]. Specifically, trials that stratified LTFs into LCFs and LPFs after PCR-correction was carried out were included in this analysis. This literature review built upon a Cochrane review of ACT medicines [3]. Regression modelling was used to explore whether factors such as age or the intensity of malaria transmission in the trial location could explain any of the variation observed in the proportion of LTFs that were symptomatic.

## Methods

### Literature review

For pre-2009 trials an existing Cochrane systematic review of clinical trials of ACT for treatment of uncomplicated malaria was used [3]. For post-2009 data a search was conducted between 20 March and 30 March, 2019 of PubMed, Medline and Embase using the term ‘artemether–lumefantrine’, limited to publications from 1 January, 2009. Details of the search strategy can be found in the Additional file 1. Results were limited to randomized controlled trials using the pre-set Ovid filters in each database. Search results were not restricted by language or geographical location. Grey literature, in particular conference abstracts, were also reviewed using results retrieved from this search.

### Inclusion criteria

Included studies were clinical trials of AL for treatment of uncomplicated malaria which reported PCR-corrected treatment failure rates and whether these failures were clinical or parasitological (i.e., asymptomatic). Only studies which used the standard dosing of AL were included: 6 doses taken over 3 days of tablets comprising 20 mg of artemether and 120 mg of lumefantrine. The number of tablets per dose is determined by weight, with one, 2, 3, and 4 tablets for individuals weighing 5–15 kg, 15–25 kg, 25–35 kg, and > 35 kg, respectively [2]. Any deviation from this regimen could lead to either enhanced or diminished drug concentrations, which could influence the time required for a recrudescent infection to become detectable by microscopy or for symptoms to return. It was also required that the trial involved only participants with a confirmed *P. falciparum* infection, who sought treatment for symptoms of malaria.

Studies that did not report any recrudescences (i.e., no LTFs after results had been PCR corrected) were excluded. Studies in which multiple drug therapy was evaluated but results not sufficiently stratified by therapy were also excluded.

From these studies, data for the number of subjects enrolled, the number and type of recrudescences observed, the duration of follow-up, and the location of the trial was extracted. Information on the inclusion criteria (if any were reported) for baseline parasite density in each trial was also extracted, as was the age range eligible for inclusion in each trial.

Titles and abstracts were reviewed against the inclusion criteria. Any discrepancies regarding inclusion/exclusion of an article was agreed upon with the consultation of fellow reviewers.

### Estimates of transmission intensity in each trial site

Estimates were used for falciparum malaria prevalence by microscopy in children between 2 and 10 years of age (*Pf*PR_2-10_) by the Malaria Atlas Project (MAP) at the time and location of each trial [22]. The R package malaria Atlas [23] provides prevalence estimates once the desired year, longitude and latitude are provided. For each site location and year of trial, this R package was used to extract prevalence estimates, averaged over a 20-km area. The latitude and longitude of each trial site was obtained using the website www.latlong.net [24].

### Statistical analysis

Bayesian regression modelling was used to explore the variation observed in the proportion of recrudescences for which symptoms returned, which is denoted by $\rho$. One advantage of a Bayesian approach is that the sampling variation in each of the original studies can be explicitly included, using information on the sample size in each study, rather than approximating these with, for example, inverse variance weights. This aids the accurate propagation of uncertainty for the final results. In a trial with $N_{tot}$ recrudescences, the number of these for which symptoms return ($N_{C}$) follows a binomial distribution, i.e. $N_{C} \sim Bin\left( {N_{tot} ,\rho } \right)$. A logistic regression framework was used to model $\rho$. Covariates included in the model were: the transmission intensity (x_1_) at the time and location of each trial (expressed in terms of the MAP-estimated *Pf*PR_2-10_), the duration of follow-up in each trial (x_2_), the age range of the cohort (x_3_), and the minimum baseline parasite density that was required for patients to be included in the original trial (x_4_). For the model containing all 4 variables, the regression model is written as

$$\text{log}\left( {\frac{{\rho_{j} }}{{1 - \rho_{j} }}} \right) = \alpha + \mathop \sum \limits_{i = 1}^{4} \beta_{i} x_{ij} , j = \left( {1, \ldots ,N} \right),$$

where the subscript $j$ enumerates the clinical trials and $\rho$ has been transformed to the log-odds scale. The continuous value for *Pf*PR_2-10_, expressed as a proportion, is retained in the regression models, and 3 categorical variables for x_2_, x_3_ and x_4_ are constructed. For duration of follow-up, the categorical variable was set to 1 for trials with a follow-up greater than 28 days and set to 0 otherwise. For age of participants, of particular interest were studies that only included young children, so x_3_ was set equal to 1 for trials that enrolled participants with a maximum age of 72 months or less, and equal to 0 for all other trials. For baseline parasite density, a categorical variable was set equal to 1 for trials that allowed individuals with a baseline parasitaemia of $\ge$ 2 × 10^5^ parasites per µl to be enrolled, and equal to 0 otherwise. The intercept, $\alpha$, represents the log-odds of a recrudescent infection being symptomatic in the baseline group, i.e. $x_{1} = x_{2} = x_{3} = x_{4} = 0$.

The regression models were fitted in R using RStan [25], via the rethinking package [26]. Due to a lack of information from previous studies to inform the parameter values, prior information was not incorporated into the fitting procedure, although weakly informative priors were used to ensure regularization [27]. Specifically, all parameters (including the intercept) were assigned a normal distribution with a mean of 0 and a standard deviation of 1.5 for their prior distributions. The goodness of fit of candidate models, containing all or some of these variables, were compared using the Watanabe Akaike Information Criterion (WAIC). The WAIC evaluates a model’s fit to the data across the whole posterior distribution, rather than just at its mode, and penalizes each model according to the number of parameters included [27]. The best fitting model has the lowest WAIC.

## Results

**Fig. 1:**
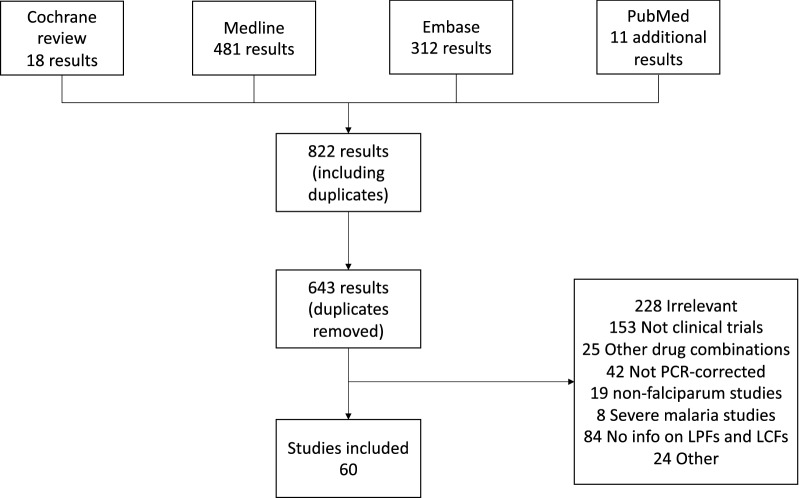
Flowchart showing the origin of all studies included in this review

The 2009 Cochrane review, artemisinin-based combination therapy for treating uncomplicated malaria [24], identified 18 studies meeting the inclusion criteria for the study. The search strategy for studies published after 2009 returned 643 results after removing duplicates (Fig. 1). Titles and abstracts were screened against the inclusion and exclusion criteria, with full texts reviewed for 335 studies. A high number of studies (84) that would otherwise have met the inclusion criteria could not be included, as LTFs due to recrudescent infections were not stratified into LCFs and LPFs. Of the 60 studies included in the analysis, several were multi-centred trials that reported results separately for each site, resulting in 75 measures of the proportion of recrudescences which were symptomatic (these studies are summarized in Additional file 2).

Across the included studies, 14 ETFs and 398 recrudescences were recorded out of 9137 patients who were enrolled and remained in the trial until at least the first day of follow-up. This results in an overall failure rate of 3.7% (95% CI (2.9–4.7%), based on a random effects meta-analysis of proportion) for this population. Of the recrudescences, 162 were recorded as symptomatic recrudescences (37.8%, 95% CIs (26.6–49.4%), based on a random effects meta-analysis of proportion). The number of recrudescences in each trial was low (median 3, range (1, 72)) The majority of studies (81%) reported PCR-corrected results at one time point, with the remaining studies (19%) reporting results at 2 time points. The most common time point (81% of studies) for reporting results was 28 days after treatment commenced, although a substantial number (33%) reported results at day 42. A number of trials enrolled only young children, especially in high transmission areas. For example, 37% of the trials included here had a maximum age for enrolment of 72 months or less. Of the studies included, there were no trials that enrolled only adults. The transmission intensity at the trial location, as estimated from the MAP, varied widely with slide prevalence in 2–10 year olds ranging from 0.2 to 86.5%. Not all studies reported information on whether they used patient inclusion criteria based on parasite densities upon presentation. For those that did, the most frequent minimum parasite density required for inclusion in a trial was 1000 (48% of trials) and 2000 (35% of trials) parasites per µl. For the maximum parasite density, 17% of trials set a value of 10^5^ parasites per µl, whilst 53% allows individuals with a baseline parasitaemia of up to 2 × 10^5^ parasites per µl to be enrolled. One study permitted patients with a baseline parasite density of up to 5 × 10^5^ parasites per µl to be enrolled.

Among the studies that only enrolled young children (aged 72 months or less), 50 out of 111 recrudescences were symptomatic (44.4%, 95% CIs (24.9, 64.6), by meta-analysis of proportion). In the remaining trials, 113 out of 287 recrudescences were symptomatic (34.3%, 95% CIs (21.1–48.3), by meta-analysis of proportion). Among studies which reported results until day 28, 104 out of 244 recrudescences were symptomatic (43.0%, 95% CIs (28.3–58.2), by meta-analysis of proportion). In the remaining studies, 59 out of 154 recrudescences were symptomatic (31.6%, 95% CIs (15.3, 48.6), by meta-analysis of proportion).

### Factors affecting the proportion of symptomatic recrudescences

Regression models were fitted to explore whether any of the variation in the proportion of recrudescences that were recorded as symptomatic could be explained by transmission intensity (represented by the estimate of malaria prevalence by microscopy in children aged 2–10, *Pf*PR_2-10_), duration of follow-up, age range of the cohort, or whether patients with very high baseline parasitaemia were enrolled in the study (Table 1). Of the 75 trials summarized in the previous sub-section, 5 were dropped from this analysis because they were carried out across multiple countries and results could not be fully stratified by location. Logistic regression models were fitted to data from the remaining 70 trials (see “Methods” for full details).

**Table 1:** Summary of trial data used for regression modelling

Quantity	Value
Total number of trials	70
Reporting days	2 trials reported on day 14, 56 trials on day 28, 22 trials on day 42, one trial on day 45, one on day 56
MAP prevalence estimates (mean and range)	25.9% (0.2%, 86.5%)
Trials treating young children only (< 72 months)	26
Total number of patients treated	8616
Patients treated in each trial (mean and range)	123 (23, 665)
Total number of patients for whom treatment failed	14 ETFs, 380 recrudescences (of which 219 were asymptomatic and 161 (42.4%) were symptomatic)

These data come from 60 published studies, splitting multi-centred studies by site (see full data in Additional file 2). This Table does not include data from 5 multi-country studies for which data could not be stratified by site (these data are included in the findings reported in Results section)

**Fig. 2:**
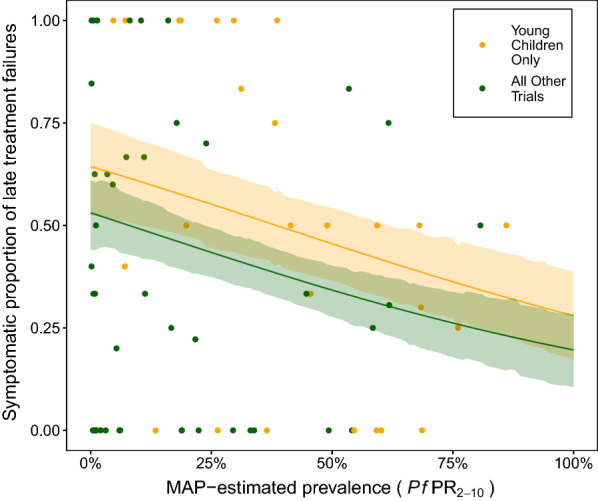
Model predictions for the proportion of recrudescences that are symptomatic. The proportion of recrudescences that are symptomatic decreases with transmission intensity, and is higher in young children. Points show data and lines are predictions from the multivariable regression modelling with 95% credible intervals as shaded areas. Results were generated from the best-fit model (Table 2), which included covariates for transmission intensity (measured as malaria prevalence) and age (binary variable, indicating trials that only enrolled children under 72 months old)

The goodness of fit for each model (containing combinations of these 4 variables) was assessed, with a penalty for the number of parameters included. The leading 5 candidate models, ordered by goodness of fit, are summarized in Table 2. The best-fit model contained 2 of the 4 covariates, those for transmission intensity and age (Fig. 2). It was found that a lower proportion of recrudescences are symptomatic in settings with a high malaria prevalence. The model predicts a 1.17-fold reduction (95% CIs 1.08–1.26) in the odds of symptoms returning in recrudescent infections for every 10% increase in prevalence. In this model, trials that enrolled only young children are expected to have a higher proportion of symptomatic recrudescences, increasing the odds of symptoms returning by 1.61-fold (95% CIs 1.01–2.59). Here the model is illustrated with an example: in a trial that enrolled only young children, the model predicts that 60.9% (95% CIs 46.9–72.4%) of recrudescences would be symptomatic if carried out in a location with a *Pf*PR_2-10_ of 10%, compared with 45.8% (95% CIs 36.5–54.5%) if the same trial was carried out in a location with a *Pf*PR_2-10_ of 50%.

**Table 2:** Factors associated with the proportion of recrudescences that were symptomatic

Variable(s) included and regression parameter(s)	WAIC	Akaike Weight	Parameter values (95% CI)	Odds ratios (95% CI)
*Pf*PR2-10 ($\beta_{1}$), per 10% increase Only young children enrolled (≤ 72 months) ($\beta_{3}$)	*506.5*	0.25	$\beta_{1}$ = − 0.154 (− 0.230, − 0.075) $\beta_{3}$ = 0.48 (0.01, 0.95)	0.86 (0.79, 0.93) 1.62 (1.01, 2.59)
*Pf*PR2-10 ($\beta_{1}$), per 10% increase Follow-up duration > 28 days ($\beta_{2}$), Only young children enrolled ($\beta_{3}$)	506.9	0.20	$\beta_{1}$ = − 0.159 (− 0.239, − 0.08) $\beta_{2}$ = − 0.26 (− 0.69, 0.16) $\beta_{3}$ = 0.48 (0.00, 0.94)	0.85 (0.79, 0.92) 0.77 (0.50, 1.17) 1.62 (1.00, 2.56)
*Pf*PR2-10 ($\beta_{1}$), per 10% increase Only young children enrolled ($\beta_{3}$), High density infections enrolled ($\beta_{4}$) (baseline parasitaemia $\ge 2 \times 10^{5}$ parasites/µl)	507.6	0.15	$\beta_{1}$ = − 0.174 (− 0.265, − 0.076) $\beta_{3}$ = 0.44 (− 0.04, 0.93) $\beta_{4}$ = 0.24 (− 0.41, 0.86)	0.84 (0.77, 0.93) 1.55 (0.96, 2.53) 1.27 (0.66, 2.36)
*Pf*PR2-10 ($\beta_{1}$), per 10% increase Follow-up duration > 28 days ($\beta_{2}$), Only young children enrolled ($\beta_{3}$), High density infections enrolled ($\beta_{4}$)	507.9	0.12	$\beta_{1}$ = − 0.180 (− 0.274, − 0.082) $\beta_{2}$ = − 0.27 (− 0.7, 0.15) $\beta_{3}$ = 0.44 (− 0.04, 0.93) $\beta_{4}$ = 0.26 (− 0.40, 0.90)	0.84 (0.76, 0.92) 0.76 (0.5, 1.16) 1.55 (0.96, 2.53) 1.30 (0.67, 2.46)
*Pf*PR2-10 ($\beta_{1}$) per 10% increase	508.8	0.08	$\beta_{1}$ = − 0.134 (− 0.208, − 0.058)	0.87 (0.81, 0.94)

A number of uni- and multivariable regression models were fitted to the data and compared. The variables included were: the MAP-estimated malaria prevalence in children 2–10 years of age at the time and location of each trial (*Pf*PR_2-10_); the duration of follow-up in each trial; age range of the cohort enrolled; high-density infections included in the trial. The model fit was assessed by WAIC. The model that included variables for malaria prevalence and the age range of the cohort provided the best fit to the data. In addition, some of the other candidate models, ordered by WAIC, and the Akaike weight associated with each model in the ensemble, are described here. Parameter values and corresponding odds ratios are summarized by the posterior means and the 95% credible intervals

As indicated by Table 2, it should be noted that the best-fit model only narrowly out-performed some of the other candidates. As a sensitivity analysis, output was also generated from an ensemble of regression models, weighted by the models’ Akaike weights [27], indicating each model’s contribution to the ensemble (Table 2). The generated model predictions (Additional file 1: Fig. S1) are very similar to those shown in Fig. 2, with slightly wider credible intervals (see caption of Additional file 1: Fig. S1, where the worked example carried out in the previous paragraph is repeated). Of the univariate models, it should be noted that only the model that included *Pf*PR_2-10_ outperformed the intercept-only model (the WAIC score of the latter was 520.1).

## Discussion

This work carried out a systematic review of clinical trials in which AL was used to treat uncomplicated falciparum malaria. Among individuals whose infections recrudesced following treatment, it was found that 37.8% (95% CIs (26.6–49.4%)) were classified as symptomatic recrudescences. An association was found between a higher proportion of recrudescences being symptomatic and younger age as well as lower transmission settings, consistent with lower prior exposure and immunity in these study populations. The number of recrudescences observed in an individual trial is usually very low (here, the median number was 3, range (1, 72)), which means that the results presented here could only be obtained via a pooled analysis.

The lack of recurrent symptoms in the majority of patients when an infection recrudesces suggests they would be unlikely to seek further treatment despite having persistent parasitaemia. These parasites have passed through drug pressure, giving any parasites with newly emerging or spreading drug resistant mutations a survival advantage. It is not known how long these recurrent infections would last, but untreated infections are often chronic and last on average about 6 months [28], potentially conferring a large transmission advantage on surviving parasites. Such a process might be particularly important during the early stages of drug-resistance evolution. Malaria parasites often acquire drug-resistance mutations in a step-wise fashion [29], with a first mutation conferring only a low level of resistance, followed by additional mutation conferring greater resistance (although few parasites are ever 100% resistant to a drug). Now that combination therapy is the norm for malaria treatment, the parasites must also acquire resistance to both drugs. When a parasite is only partially resistant, a second course of treatment in patients with recurrent symptomatic treatment may be enough to clear all the parasites (particularly if a different drug is given, e.g., an ACT with a different partner drug). Therefore, understanding the probability of re-treatment after initial recrudescence is important to quantifying the potential spread of resistance, for example in mathematical models [30].

One interesting avenue for further work would be to repeat the analysis for another ACT. In particular, it would be interesting to see if different results were obtained for dihydroartemisinin-piperaquine, as piperaquine has a much longer half-life than lumefantrine [31], and might be expected to suppress recrudescent infections for a longer period of time thereby delaying the reappearance of symptoms. To understand the role of recurrent symptoms in the spread of drug resistance, it would also be useful to collect similar data from trials where drug resistance is present.

One limitation of using this approach to estimate the proportion of recrudescences that become symptomatic is that it is restricted by the duration of follow-up in each trial. One cannot be sure if a patient would become symptomatic beyond the end of the follow-up period. Equally, an asymptomatic infection detected during follow-up and treated within the trial setting may have later developed into a symptomatic infection in a non-trial setting. Within the follow-up period, some trials will assess patients regularly (e.g., every week), whereas other trials will assess the patients at the end of the evaluation period. In a trial in which patients are assessed more frequently, recrudescent infections will be detected earlier, which could prevent some of them from becoming symptomatic. In the regression modelling, no association was found between the duration of follow-up and the likelihood of symptoms returning after treatment failed. However, the dataset has very little temporal resolution (83% of studies had only reported results for one time point), which made it difficult to scrutinize this in any detail. Outside trial settings, AL failure rates are likely to be somewhat higher due to imperfect adherence to treatment [32, 33]. It is possible that the likelihood of symptoms returning upon recrudescence of parasitaemia would be different in patients who did not fully adhere to the treatment regimen, e.g., due to parasitaemia recrusdescing earlier, due to diminished lumefantrine concentrations.

Not all studies provided information on the inclusion criteria for baseline parasite density in each trial. For studies that did not report it, it was assumed that any individual with uncomplicated symptoms who was positive for parasites by microscopy could be enrolled in the trial. Furthermore, the connection between the range of parasitaemia permitted in each trial and the distribution of parasitaemia measured in the cohort is not a clear one, but this was not available for all trials either.

In recent within-host modelling work [15, 32] the link between poor adherence and treatment failure was investigated, with the more recent study translating treatment failure into an enhanced capacity to transmit malaria. It was shown that the proportion of recrudescent infections that are retreated had a big impact on onward transmission [15], but no information was available as to what, in a particular setting, this proportion would be. In reality, this will depend on many factors, not least the availability and affordability of a second course of treatment. However, outside of a trial setting it is very unlikely that an asymptomatic recrudescent infection would be detected and retreated.

## Conclusion

In this study, by pooling together data from a large number of clinical trials, insight was gained into the likelihood of patients becoming symptomatic again following recrudescence of a falciparum malaria infection. It was found that this is more likely in younger children and in lower transmission settings.

## Supplementary information

**Additional file 1.** Supplementary Figure 1 and details of the search strategy for the systematic review.

**Additional file 2.** Details of the studies included in the systematic review.

## Data Availability

All the data extracted from the publications included in the systematic review are included in Additional file 2, as an Excel file.

## Abbreviations

ACT: Artemisinin-based combination therapy
AL: Artemether–lumefantrine
PCR: Polymerase chain reaction
ETF: Early treatment failure
LTF: Late treatment failure
LCF: Late clinical failure
LPF: Late parasitological failure
MAP: Malaria Atlas Project
*Pf*PR_2-10_: *Plasmodium falciparum* prevalence in children aged 2–10 years
WAIC: Watanabe-Akaike Information Criterion
OR: Odds ratio
