# Supplementary material for: Asymptomatic recrudescence after artemether–lumefantrine treatment for uncomplicated falciparum malaria: a systematic review and meta-analysis
Source: Malar J. 2020 Dec 9;19:453. doi: 10.1186/s12936-020-03520-1 (PMC7724891; doi:10.1186/s12936-020-03520-1)
Supplement: Supplementary file 1 — Additional file 1. Supplementary Figure 1 and details of the search strategy for the systematic review. [file 12936_2020_3520_MOESM1_ESM.pdf]

Asymptomatic recrudescence after artemether-lumefantrine treatment for uncomplicated falciparum malaria: a systematic review and meta-analysis

Rida Mumtaz[1], Lucy C. Okell[2], Joseph D. Challenger[2 *]

1. Faculty of Medicine, Imperial College London, London, United Kingdom

2. Medical Research Council Centre for Global Infections Disease Analysis, Department of Infectious Disease Epidemiology, Imperial College London, London, United Kingdom

**Corresponding Author. Email: j.challenger@imperial.ac.uk*

SUPPLEMENTARY MATERIALS

This document contains:

1. **Supplementary Figure 1**
2. **Details of the search strategy used for the systematic review**

Supplementary Figure 1: The proportion of treatment failures which are symptomatic increases with lower transmission intensity and younger age. Points show data, and lines are predictions from the multivariable regression modelling with 95% credible intervals as shaded areas. In Figure 2 of the main text, we showed results generated from the model that provided the best fit (see Table 2). For comparison, we here show results generated from the ensemble of regression models, with models weighted according to their goodness of fit (see Table 2). For the two variables not displayed in the figure (follow up period and parasitaemia cut-off for enrolment) we generated results for trials with a 28-day follow-up period and a parasitaemia cut-off of <$2x10^5$ parasites per microlitre. However, these two variables do not strongly influence the results obtained. To facilitate comparison of the results generated from the model ensemble with those generated from the best performing model, we here show the results obtained for the illustrative scenario outlined in the Results section. In a trial that only enrolled young children, the model ensemble predicts that 58.9% (95% CIs, 45.8-71.1%) of late treatment failures would be symptomatic if carried out in a location with a *Pf*PR_{2-10} of 10% compared with 43.2% (95% CIs, 30.0-55.1%) if the same trial was carried out in a location with a *Pf*PR_{2-10} of 50%.

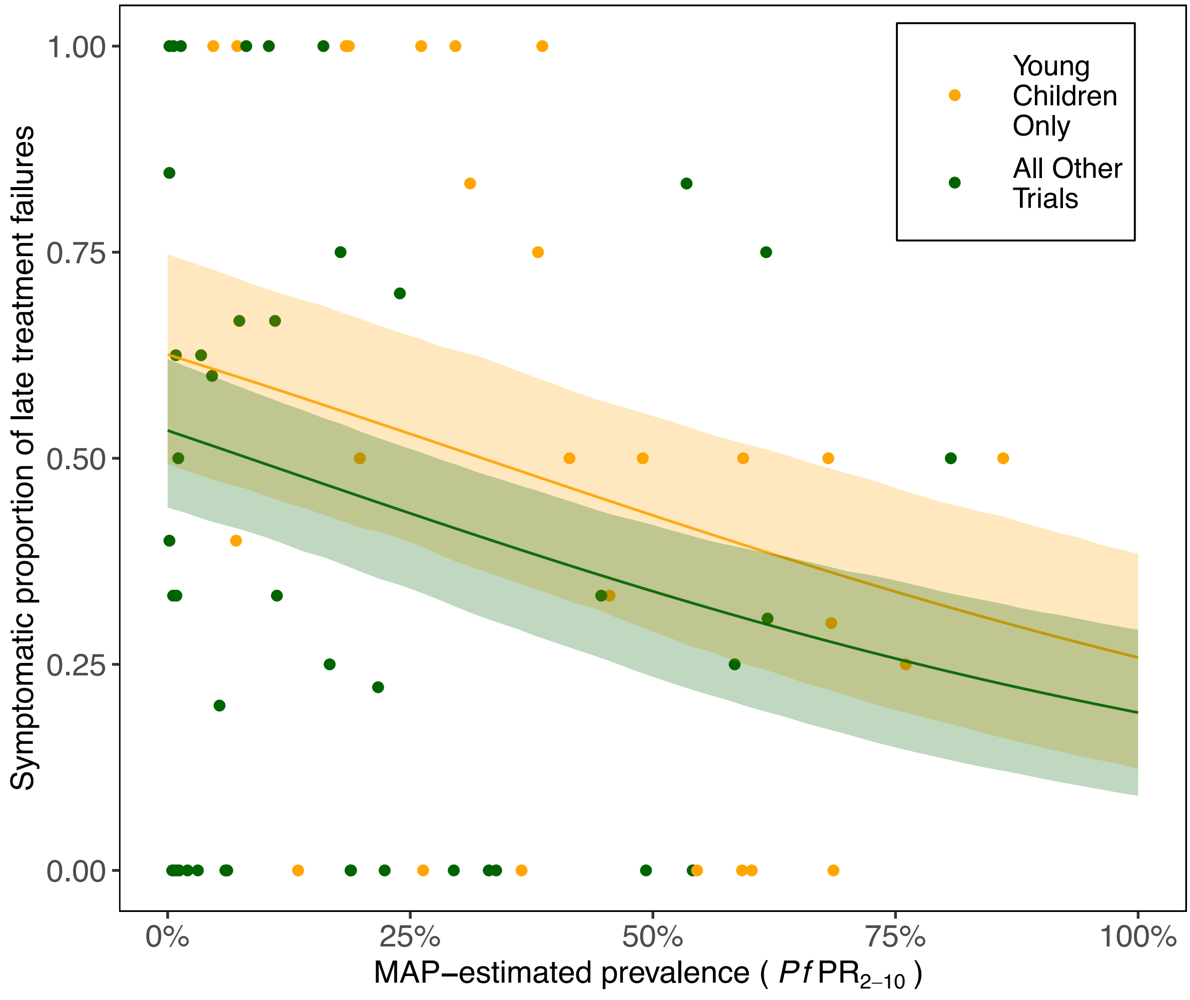

Details of the search strategy used for the systematic review

artemether plus benflumetol/	(artemether adj2 lumefantrin*)	(artemether adj2 benflumetol)	cgp 56697	cgp56697	co artem*	coartem*
coa 566	coa566	riamet	Blackwater fever.	(falciparum adj2 infection)	falciparum malaria	malaria tropic*
P?falciparum infection	P?falciparum malaria	pernicious malaria	plasmodium falciparum infection	plasmodium falciparum malaria.		

Table 1- Search Terms Embase

clinical trial.de.	randomized controlled trial.de.	randomization.de.	single blind procedure.de.
double blind procedure.de.	crossover procedure.de	placebo.de.	prospective study.de.
(randomi?ed controlled adj1 trial*).mp.	rct.mp.	(random* adj1 allocat*).mp.	(single adj1 blind*).mp
(double adj1 blind*).mp.	((treble or triple) adj1 (blind* or placebo*)).mp		

Table 2- Ovid Filter for Randomised Controlled Trials in Embase

Artemether, Lumefantrine Drug Combination/	artemether plus benflumetol.mp.	(artemether adj2 lumefantrine).mp.	cgp 56697	cgp56697
co artem*	coartem*	riamet.	(falciparum adj2 malaria)	(falciparum adj2 infection)
falciparum malaria	exp Malaria, Falciparum	blackwater fever	malaria tropic*	P?falciparum
p falciparum	Plasmodium falciparum	p?falciparum infection	p?falciparum malaria.	pernicious malaria.
plasmodium falciparum infection	plasmodium falciparum malaria			

Table 3- Search Terms Medline

randomized control trial.mp.	controlled clinical trial.mp.	randomi?ed.mp.	placebo.mp.
drug therapy.mp.	randomly.mp.	trial.mp.	groups.mp.

Table 4- Ovid Filter for Randomised Controlled Trials In Medline
